# The trabecula septomarginalis (Leonardo’s cord) in abnormal ventriculo-arterial connections: anatomic and morphogenetic implications

**DOI:** 10.1186/1749-8090-9-71

**Published:** 2014-04-21

**Authors:** Athos Capuani

**Affiliations:** 1 Paediatric Hospital Gatien de Clocheville CHRU Tours, Paediatric Cardiac Surgery, 49 Boulevard Béranger, 37044 Tours cedex 9, France

**Keywords:** Ventriculo-arterial connections, Trabecula septomarginalis, Infundibular septum, Ventricular septum, Ventriculo-infundibular fold

## Abstract

**Background:**

The abnormal ventriculo-arterial connections in atrio-ventricular concordance and situs solitus with two well developed ventricles include the range from tetralogy of Fallot throughout the different forms of double outlet right ventricle to transposition of great arteries.

The infundibular septum and the trabecula septomarginalis are the fundamental anatomical landmarks for the segmental analysis.

In these abnormalities there is a pathological progressive counter-clockwise rotation of the infundibular septum which divorces from the antero-superior limb of the trabecula septomarginalis and achieves his identity. Is there any anatomical evidence of a simultaneous abnormal counter-clockwise rotation of the trabecula septomarginalis?

**Methods:**

Malposition of great arteries is a generic term since all relationships have to be expected.

We present specimens with anatomical evidence of a progressive counter-clockwise rotation from 0° to about 180°of the plane passing throughout the trabecula septomarginalis’s limbs.

**Results:**

We can observe sequentially:

1. Malformations in which the posterior limb of the trabecula septomarginalis is committed to the ventriculo infundibular fold: (tetralogy of Fallot, double outlet right ventricle with sub-aortic ventricular septal defect, truncus arteriosus and doubly committed ventricular septal defect);

2. Malformations in which the posterior limb of the trabecula septomarginalis is committed to the infundibular septum (double outlet right ventricle with sub-pulmonary ventricular septal defect, transposition of great arteries).

**Conclusions:**

1. The sequential-segmental analysis identify all the morphologies.

2. The trabecula septomarginalis plane presents a progressive counter-clockwise twist on the long axis.

3. Since the trabeculated portions of the ventricles are the oldest developmental components, our observations support the hypothesis that the abnormal ventriculo-arterial connections could be in relation with a pathological myocardial process during early cardio-genesis.

We are promoting new studies to investigate our anatomical observations.

## Background

During embryogenesis the junction of the myocardial outflow tract with the great arteries undergoes remodelling [1].

A counter-clockwise rotation of the infundibular septum (IS) [2] from tetralogy of Fallot (TF) to different forms of double outlet right ventricle (DORV) to transposition of great arteries (TGA) has been described [3-5].

Is there any anatomical evidence of a sequential counter-clockwise rotation at ventricular level?

**Figure 1:**
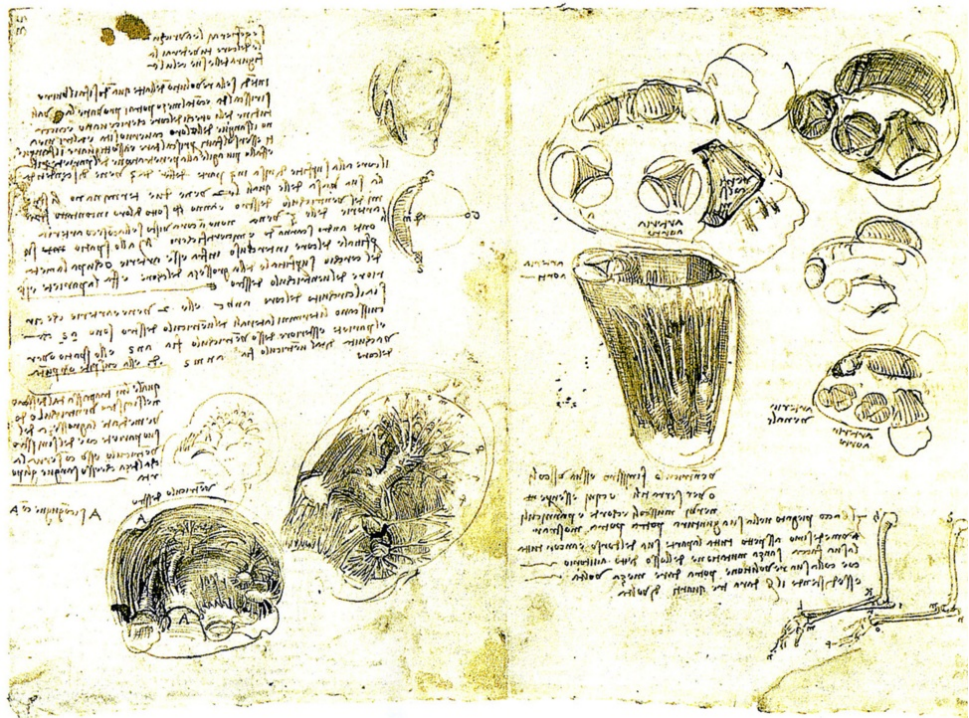
**TSM and right ventricle.** Drawing from Leonardo da Vinci.

The trabecula septomarginalis (TSM) [6] first observed by Leonardo da Vinci in 1513 [7] (Figure 1, Leonardo’s cord) is formed by compaction of the apical trabeculations on the septal surface of the right ventricle (RH Anderson, personal communication 2012).

Since the trabeculated portions of the ventricles are the oldest developmental components [8] they form the basis on which malformations of the inlet or the outlet, or both, are superimposed.

We examined the relationships between the IS and the TSM in specimens representing sequentially the spectrum of abnormal ventriculo-arterial connections in situs solitus.

## Methods

For the terminology refer to Anderson et al. and Restivo et al. [9,10].

In previous studies we identified the IS and the TSM as fundamental anatomical landmarks in abnormal ventriculo-arterial connections [11-15]. We reviewed the specimens previously collected [11,16,17] and our recent observations.

## Results

**Figure 2:**
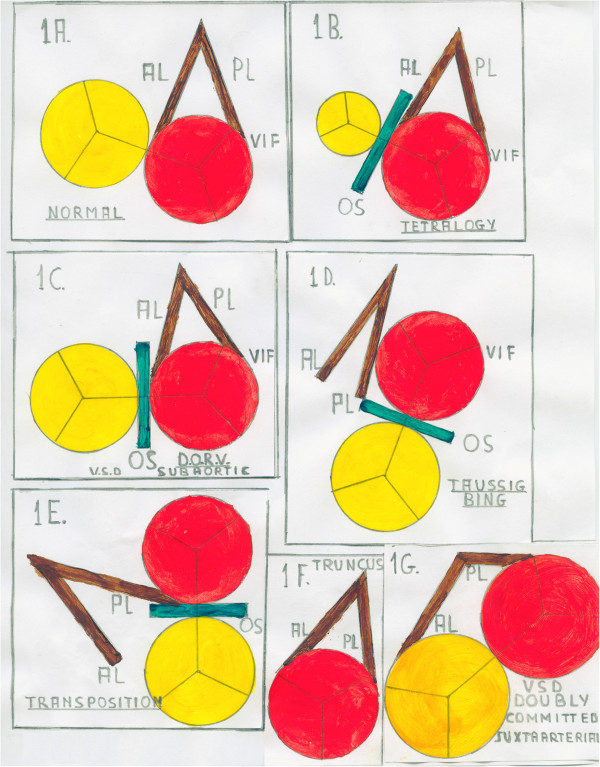
**Conceptual relationships between IS and VIF in abnormal ventriculo-arterial connections in situs solitus and atrio-ventricular concordance. 1A** Normal relationships. **1B** TF. Anterior deviation/displacement of the IS (compare to Figure 4). **1C** DORV with sub-aortic VSD. The AL blends or is committed to the counter-clockwise twisted IS. The PL blends or is committed to the VIF (compare to Figure 5). **1D** Taussig-Bing (DORV with sub-pulmonary VSD). The PL blends or is committed to a more counter-clockwise twisted IS (compare to Figure 8). **1E** TGA. The IS inserts abnormally to the TSM forming a totally displaced infundibulum (compare to Figure 9). **1F** and **1G** Absent IS in truncus arteriosus and doubly committed juxta-arterial VSD. The PL still blends or is committed to the VIF of the single outlet or of the aorta (compare to Figure 7 and 6).

In Figure 2 we drew the progressive counter-clockwise rotation of the IS and TSM and the relationships between the TSM’s limbs (anterior limb: AL, posterior limb: PL) and the ventricular septal defect (VSD).

We may imagine a pre-established appointment between the IS and the plane passing through the limbs of the TSM conceptually representing the ventricular septum (VS).

Standing on the base of the heart facing down we can observe an abnormal progressive counter-clockwise rotation of the IS and the VS plane on the long axis.

**Figure 3:**
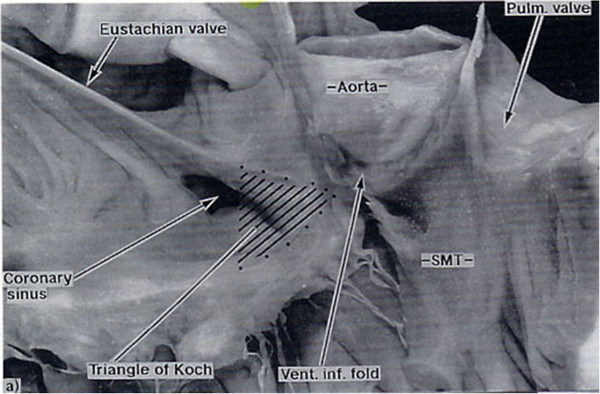
**Normal heart.** The roof of the right ventricle has been removed. The VIF (inner curvature) forms the greatest part of the supraventricular crest. The PL of the TSM blends with the VIF.

In the normal heart the IS and the VS are well aligned: the AL blends with the IS (Figure 2A, compare with Figure 3).

In the spectrum of abnormal ventriculo-arterial malformations the IS progressively divorces from the TSM creating an angle at the insertion to the AL.

**Figure 4:**
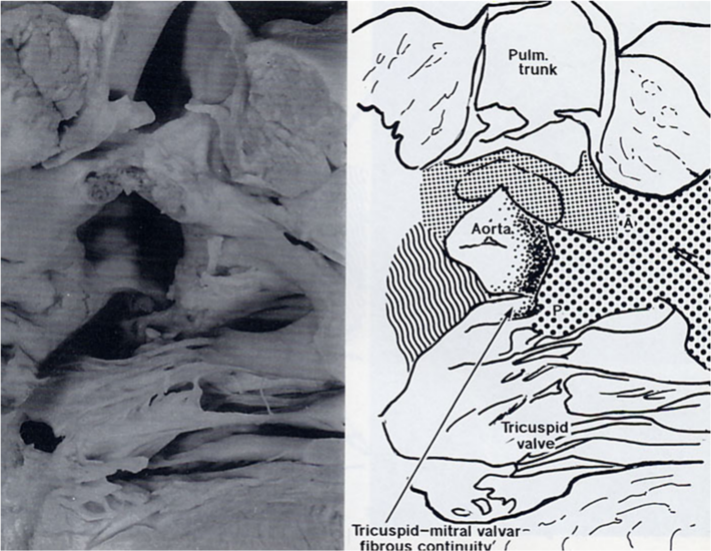
**TF with pulmonary atresia.** The IS is deviated anteriorly and is attached to the AL of the TSM as a free standing structure producing pulmonary atresia. The PL is committed to the VIF which stops short of the PL and there is tricuspid-aortic continuity.

**Figure 5:**
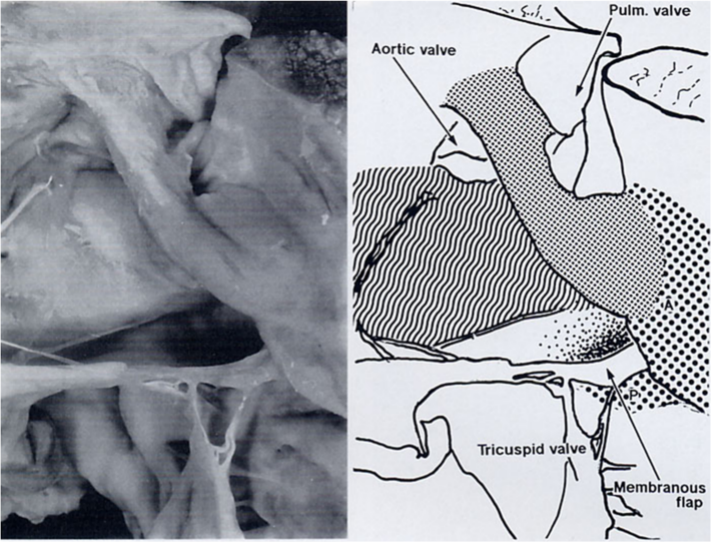
**DORV with sub-aortic VSD.** The PL is committed to the VIF. The AL blends with the IS. The VIF is well represented creating a complete muscular sub-aortic infundibulum.

With an angle from 0° to about 90° the aorta becomes dextro-posed and more anterior. We observe sequentially dextro-position of the aorta, TF (Figure 2B, compare with Figure 4), DORV with sub-aortic VSD (Figure 2C, compare with Figure 5).

The VSD represents a malalignment gap between VS and IS, is in sub-aortic position and is cradled between the limbs of the TSM.

**Figure 6:**
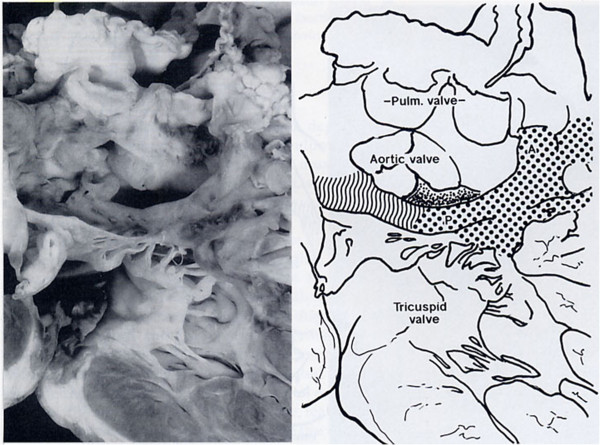
**Doubly committed juxta-arterial VSD.** The VSD opening is between the leaflets of the arterial valves and is roofed by fibrous continuity between the arterial valves.

**Figure 7:**
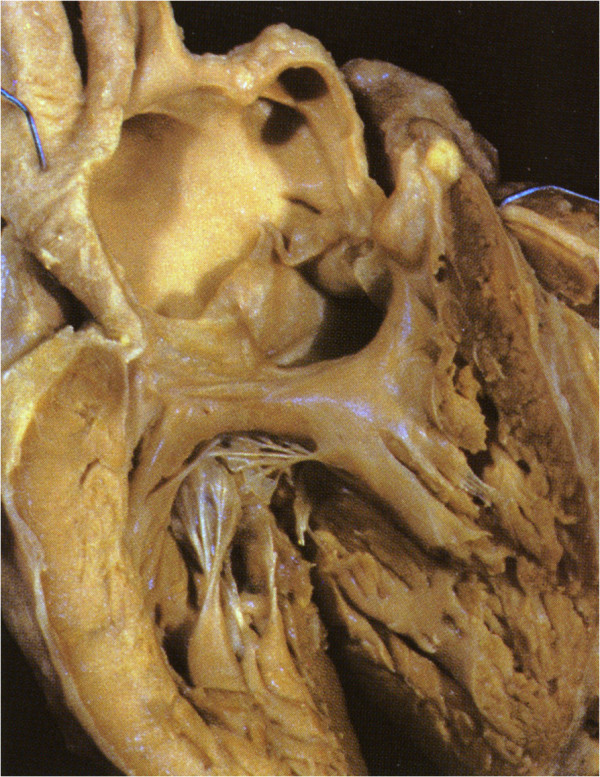
**Truncus arteriosus.** The PL blends with the VIF forming a muscular postero-inferior rim of the VSD.

The VSD may also be doubly committed juxta-arterial (Figure 2G, compare with Figure 6) in absence of IS or in the settings of truncus arteriosus (Figure 2F, compare with Figure 7).

In these sequence the AL blends or is committed to the deviated IS.

**Figure 8:**
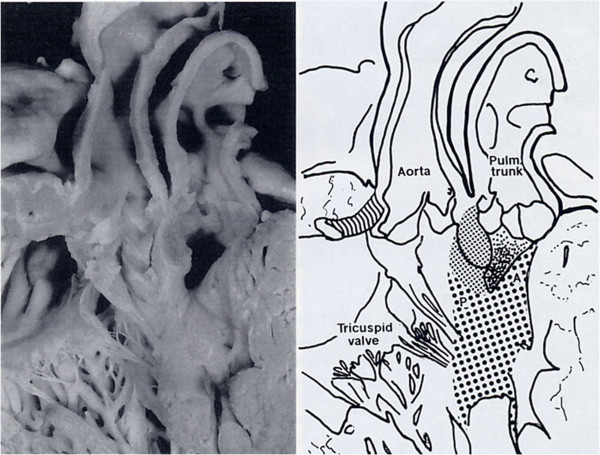
**DORV with sub-pulmonary VSD (Taussig-Bing).** The IS fuses with the VIF rather than the AL creating a sub-aortic infundibulum. The PL blends with the VIF. The AL is displaced in a cephalic position.

As the counter-clockwise rotation of the VS plane increases to about 180°, is the PL which blends or is committed to the IS, the VSD becomes sub-pulmonary (Figure 2D, compare with Figure 8) and we observe the Taussig-Bing spectrum.

At the end of the VS rotation, at about 180°, there is alignment of the IS with the VS as in the normal heart but the IS is abnormally inserted to the TSM.

**Figure 9:**
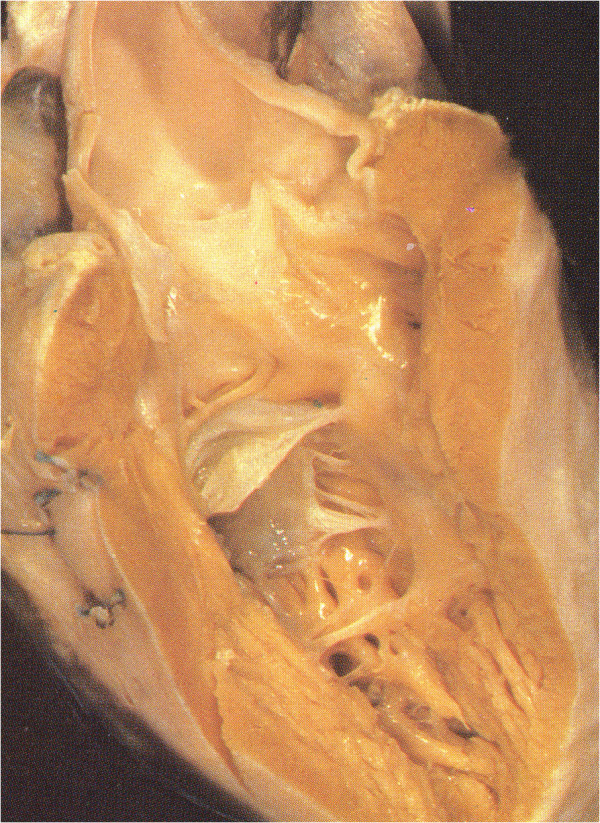
**TGA. Sub-aortic infundibulum.** The limbs of the TSM are about 180° twisted in a cephalic position.

The aorta arises from the right ventricle with a sub-aortic infundibulum and there is pulmonary-mitral fibrous continuity normally present in classic TGA with intact septum. (Figure 2E, compare with Figure 9).

**Figure 10:**
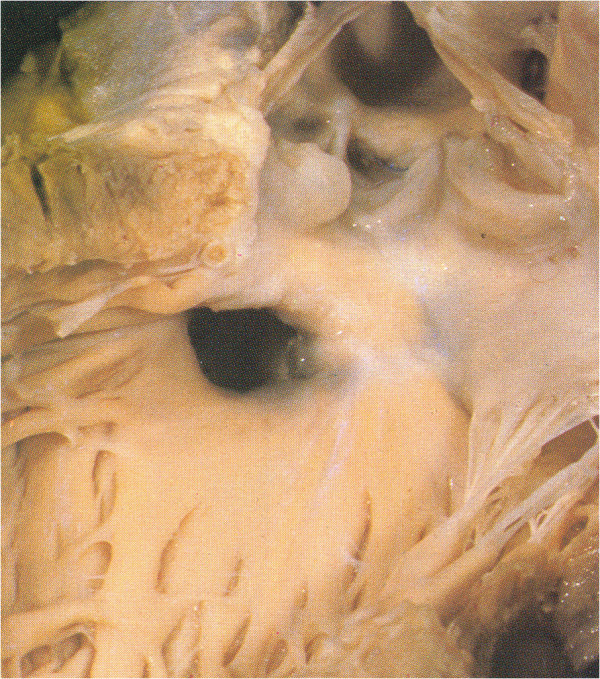
TGA with sub-pulmonary obstruction due to posterior deviation of the IS.

In these settings if the IS and the TSM plane are not well aligned there is outlet-ventricular malaligned VSD. The IS may be displaced posteriorly with sub-pulmonary obstruction (Figure 10).

**Figure 11:**
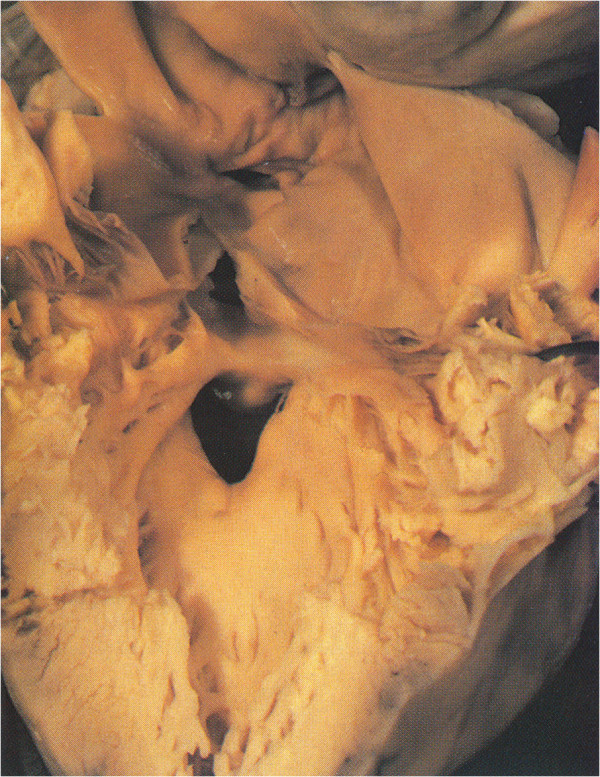
Posterior deviation of the IS in a heart with concordant ventriculo-arterial connections and interruption of the aortic arch.

These sub-arterial settings are comparable to those observed in concordant ventriculo-arterial connections with posterior deviation of the IS and sub-aortic stenosis (Figure 11).

## Discussion

The cardiac malformations with abnormal ventriculo-arterial connections in situs solitus and atrio-ventricular concordance include the TF throughout the different forms of DORV to TGA.

In this sequence there is partial or complete loss of the outflow spiralling flow pattern, gradual loss of the normal aorto-mitral continuity and progressive development of pulmonary-mitral continuity.

In these abnormalities several different surgical procedures can be applied depending upon the specific morphological sub settings. The pre existing morphology clearly affects the surgical strategy [18].

The anatomy is the prerequisite to avoid all ambiguities [19] by giving in each case the sequential-segmental analysis [20,21].

The ventricular outflow tract arises from a recently discovered second source of myocardial cells [22-24]: there is involvement of mesenchymal tissues derived from endocardium, mesoderm and migrating neural crest cells [25].

The ventriculo-arterial connections are achieved by adhesion of the fused and muscularised proximal outflow cushions to the primary muscular ventricular septum [26].

Early in development the largest part of the myocardial wall shows a trabecular arrangement similar to the abnormal pattern described as ventricular non compaction [27]. Since the trabeculated portions of the ventricles are the oldest developmental components [8] the abnormal ventriculo-arterial connections could be in relation with a non compaction myocardial process resulting in a twisted VS and finally in the spectrum of malformations we are observing.

To describe the different morphologies malposition of the great arteries is a generic term since the variability of the arterial relationships is considerable and all relations from normal to side by side to antero posterior have to be expected [15].

It is better to analyze the outflow in terms of three components: the intra pericardial trunks, the arterial valves, and the ventricular tracts [28].

In the normal heart the sub-pulmonary supra-ventricular crest is made up of the muscularised proximal outflow cushions (RH Anderson personal communication 2012). Most of this muscle becomes the free-standing sub-pulmonary infundibulum and a small part persists as real muscular IS but in the normal heart cannot be recognised (Figure 3).

The key feature of the morphology in abnormal ventriculo-arterial connections is the location and insertion of the IS relative to the remainder of the VS [11].

The TSM, first drawn by Leonardo da Vinci in 1513 (Figure 1), is an extensive septal trabeculation of the right ventricle formed by compaction of the apical muscular trabeculations on the septal surface (RH Anderson personal communication 2012).

The TSM is formed by an antero-superior limb and an infero-posterior limb and the plane passing throughout the limbs conceptually may represent the plane of the VS.

At Carnegies stages 15-19 the outflow tract makes a marked counter clockwise rotation [1] and in mouse models by transgenic studies in mutant embryos with cono-truncal defects has been reported a counter clockwise rotation of the outflow suggesting a myocardial perturbation [29].

Indeed in the spectrum of abnormal ventriculo-arterial connections from TF to DORV to TGA we can observe a sequential right-left counter clockwise rotation of the IS facing down the ventricles standing on the base of the heart and a simultaneous progressive twisting right-left of the VS plane on the longitudinal axis.

In TF (Figure 4) there is dextro-anterior deviation of the IS [30] and the angle between the IS and the VS has been reported from 60° up to 130°. In the majority of cases is about 90° [2]. The PL committed to the ventriculo infundibular fold (VIF) follows the dextroposition of the aorta and the AL blends with the IS twisting the TSM.

If the IS is absent the VSD becomes doubly committed juxta arterial: the PL still blends with the VIF (Figure 6).

A similar disposition of the limbs can be found in the common arterial trunk (Figure 7).

In this morphology the PL may form a muscular postero-inferior rim on the VSD or there may be a fibrous continuity tricuspid-truncal valve.

In DORV the counter-clockwise rotation is more accentuated (between 90° and 180°) [31-33].

The VSD represents a malalignment gap between the VS and the IS and may be committed or not committed to the great arteries. It can also be in relation with one great artery, however not directly committed owing to an extensive VIF or an extreme dextroposition of the aorta or aberrant chordae tendinee [34].

According to some investigators [2] the diagnosis of DORV should be reserved for hearts with a bilateral sub-arterial infundibulum. In contrast others formulated the concept that DORV is a malformation in which both great arteries arise completely or almost from the right ventricle with or without mitro-aortic or mitro-pulmonary continuity [31].

These different views reflect the fact that the term DORV in reality identifies different morphologies inside a spectrum of malformations.

In the settings of DORV with sub-aortic VSD the AL is still committed to the IS (Figure 5).

In DORV with sub-pulmonary VSD it is the PL which blends or is committed to the IS and the AL is displaced antero-superiorly (Figure 8).

There is a almost complete twisting of the TSM on the long axis.

We consider the overall spectrum of hearts with sub-pulmonary VSD to represent the Taussig-Bing.

This malformation has the names of the authors who described the pathology for the first time.

In the original paper the two vessels are side by side, the pulmonary artery being in its normal place [33], however as pointed out the relationships of the great arteries are variable.

There are three patterns of the angle between the IS and the remainder of the muscular VS plane: right angle (90°: great arteries side by side), acute angle (90° < 180°: aorta dextroposed and anterior), parallel (about 180°: great arteries antero-posterior) [35].

The commonest coronary pattern in the parallel position is comparable to one found in complete transposition of great arteries, what we actually expect being in these cases about at the end of VS 180° rotation.

The following morphology in the progressive TSM twist is represented by the TGA [2,36-43].

When more than half of the circumference of the pulmonary valve is supported by the left ventricle the ventriculo-arterial connections are considered discordant rather than double outlet.

The second cardiac lineage rotates as enters the heart before the outflow tract cushions are formed. In the normal cardio-genesis the cushions are spiral and the myocardium retracts as they fuse. In the settings of TGA the cushions are initially formed in straight fashion and the difference is found in the way that the distal ends of the cushions fuse with a protrusion from the dorsal wall of the aortic sac (RH Anderson personal communication 2012).

In classical TGA there is mitro-pulmonary continuity, the aorta is anterior and right sided and the aortic valve is supported by a muscular infundibulum, however, in some cases the aorta may be left sided (S, D, L transposition) [41] or posterior and right sided (normal relations) [42] and in some hearts there is a muscular sub-arterial infundibulum in both ventricles.

In TGA with intact septum the IS inserts abnormally to the TSM forming a totally displaced infundibulum (Figure 9). There is no correlation between the anterior position of the aorta and the length of the infundibulum and the typical VSD is outlet ventricular [38] however other VSD’s types as trabecular or inlet of different origin may be associated.

In transposition with aorta to the left (S, D, L) the IS was found deviated posteriorly and leftward squeezing the sub-pulmonary outflow tract with an abnormal cono-septal angulation varying from 71+/-44 degrees [41], what we expect considering that the VS rotation on the long axis according the sequence of our observations is at or close to 180° (Figure 10).

Indeed this morphology is similar to the one observed in posterior deviation of the IS in concordant ventriculo-arterial connections with VSD and sub-aortic narrowing in which obviously the VS developed normally (Figure 11).

## Conclusions

I. The counter-clockwise rotation of the IS and the simultaneous twist of the TSM characterise all sub-settings of abnormal ventriculo-arterial connections in situs solitus and atrio-ventricular concordance. This is particularly important for the diagnosis and corrective surgery in this complex spectrum of malformations.

II. The presented anatomical observations support the hypothesis that the abnormal ventriculo-arterial connections may be successive stages of the same embryo-genetic process at ventricular level and that may arise from an abnormal myocardial rotation in addition to an abnormal outflow tract septation.

III. The abnormal ventriculo-arterial connections could be in relation with a pathological myocardial compaction process. We promote further investigations.

### Limitations of the study

This study relates to abnormal ventriculo-arterial connections in situs solitus atrio-ventricular concordance and two well developed ventricles.

A further spectrum of malformations can be seen extending towards double outlet left ventricle.

## Abbreviations

DORV: Double outlet right ventricle; TF: Tetralogy of Fallot; TGA: Transposition of great arteries; IS: Infundibular septum; VS: Ventricular septum; TSM: Trabecula septomarginalis; PL: Posterior limb of the TSM; AL: Anterior limb of the TSM; VIF: Ventriculo infundibular fold; VSD: Ventricular septal defect.

## Competing interests

The Author has no financial or no financial competing interests.

This study received no specific grant from any funding agency, commercial or not-for-profit sectors.

The Author transfers the copyright to the publisher.
